# Exploratory case series of conversion to carbon ion radiotherapy after systemic therapy in advanced hepatocellular carcinoma

**DOI:** 10.1007/s13691-025-00839-x

**Published:** 2026-01-12

**Authors:** Haruka Anzai, Chihiro Miwa, Sadahisa Ogasawara, Makoto Fujiya, Hiroki Kurosaki, Takahiro Tsuchiya, Ryohei Yoshino, Keiichi Katayama, Midori Sawada, Teppei Akatsuka, Ryo Izai, Takuya Yonemoto, Sae Yumita, Keisuke Koroki, Masanori Inoue, Masato Nakamura, Naoya Kanogawa, Shingo Nakamoto, Hirokazu Makishima, Makoto Shinoto, Masaru Wakatsuki, Shigeru Yamada, Hitoshi Ishikawa, Jun Kato

**Affiliations:** 1 Department of Gastroenterology, Graduate School of Medicine, Chiba University, 1-8-1 Inohana, Chuo-Ku, Chiba, 260-8670 Japan; 2 National Institute of Radiological Sciences Hospital, National Institutes for Quantum and Radiological Sciences and Technology, Chiba, Japan

**Keywords:** Carbon ion radiotherapy, Hepatocellular carcinoma, Conversion therapy, Systemic therapy

## Abstract

Hepatocellular carcinoma (HCC) is a leading cause of cancer-related mortality, with advanced cases often presenting macrovascular invasion (MVI) or extrahepatic spread (EHS), limiting curative options. Latest systemic therapies, such as atezolizumab plus bevacizumab, have demonstrated potential in converting advanced HCC into treatable states. Carbon ion radiotherapy (C-ion RT), with its superior dose localization and biological effectiveness, has emerged as a promising treatment for advanced HCC. This retrospective exploratory case series reviewed eight patients with advanced HCC who received C-ion RT following systemic therapy at a single Japanese institution between 2021 and 2023. Data collection and follow-up were completed in October 2024. During the study period, 188 patients with advanced HCC received systemic therapy at our institution. Among these, 176 patients (93.6%) continued systemic therapy alone or received additional transarterial chemoembolization (TACE) for local disease control, while 12 patients (6.4%) were evaluated for curative-intent local therapy through multidisciplinary discussion. Patients were considered for conversion therapy after showing tumor shrinkage or loss of contrast enhancement during systemic therapy, but surgical resection was judged infeasible due to poor liver function or surgical tolerance. Therefore, C-ion RT was selected to treat all residual intrahepatic lesions. Clinical characteristics, treatment details, radiological responses, and safety outcomes were evaluated. The median age was 71 years, with 62.5% presenting MVI and 62.5% having tumors ≥ 90 mm in diameter. Systemic therapy included atezolizumab plus bevacizumab in seven cases and lenvatinib in one case. Following systemic therapy, C-ion RT achieved radiological cancer-free (rCF) status in five patients, including four with MVI and three with major portal vein invasion (Vp4). No clinically meaningful deterioration in liver function was observed during treatment, and adverse events were minimal. Patients achieving rCF maintained short-term disease control. Median follow-up was 11 months (range, 5–16). This exploratory case series suggests that C-ion RT following systemic therapy may be a feasible and safe local consolidation approach for highly selected patients with advanced HCC. C-ion RT achieved short-term local control without deterioration of liver function in this small cohort. Given the limited sample size and follow-up duration, these findings are preliminary and warrant validation in prospective studies with larger cohorts to establish its role in multidisciplinary management.

**Supplementary Information:**

The online version contains supplementary material available at 10.1007/s13691-025-00839-x.

## Introduction

Hepatocellular carcinoma (HCC) is the most common form of primary liver malignancy and represents a leading cause of cancer-related mortality worldwide [1]. Approximately 60% of cases are diagnosed at advanced stages, precluding curative options such as surgical resection or locoregional therapies [2]. The Barcelona Clinic Liver Cancer (BCLC) staging system, a globally recognized framework, classifies hepatocellular carcinoma (HCC) with macrovascular invasion (MVI) and/or extrahepatic spread (EHS) as advanced stage, for which curative treatment is considered challenging [3]. Current guidelines recommend systemic therapy as the standard first-line treatment for these patients.

The introduction of atezolizumab plus bevacizumab has shifted systemic therapy for advanced HCC toward combination immunotherapies, demonstrating unprecedented efficacy as first-line treatments [4]. These therapies have demonstrated efficacy in converting advanced HCC, particularly cases with MVI or EHM, into a potentially curable state, enabling subsequent interventions such as surgery, radiofrequency ablation (RFA), or selective transarterial chemoembolization (TACE) [5, 6]. As an extension of this concept, atezolizumab plus bevacizumab conversion (ABC) therapy has been proposed as a strategy to further improve patient outcomes by enabling subsequent curative interventions [7]. A multi-center collaborative study investigating ABC therapy demonstrated a high rate of drug-free status achievement (35%) [8]. Furthermore, previous studies have reported that achieving radiological cancer-free (rCF) status in unresectable advanced HCC is associated with significantly improved prognosis [9]. Patients who attained rCF status demonstrated a prolonged overall survival (OS), with the median OS not reached, whereas those who did not achieve rCF had a median OS of 11.7 months. This underscores the prognostic significance of rCF status in advanced HCC. Consequently, the integration of conversion therapy with conventional treatment modalities has become increasingly important in the management of advanced HCC.

Carbon ion radiotherapy (C-ion RT) is an advanced form of radiation therapy that utilizes high-energy particle beams of accelerated carbon ions. A key advantage of C-ion RT over conventional radiotherapy lies in its superior dose concentration, attributed to the Bragg peak phenomenon and high linear energy transfer (LET) characteristics [10]. These properties enhance its biological effectiveness, making it a promising treatment option for HCC. In HCC, C-ion RT has demonstrated high efficacy, with reports indicating local control rates of approximately 90% at two years post-treatment [10, 11]. Even in cases with MVI, C-ion RT achieves local control rates comparable to surgical resection while minimizing adverse events and preserving liver function [12, 13]. Given these advantages, C-ion RT has been increasingly recognized as a potential curative option for advanced HCC. In Japan, C-ion RT has been approved for clinical use and is covered by the national health insurance system, facilitating its integration into routine clinical practice.

As systemic therapies continue to improve, an increasing number of patients with advanced HCC are achieving significant tumor responses. This raises the possibility that C-ion RT at this stage could serve as a definitive therapy, ultimately aiming for a rCF status. However, clinical evidence on the use of C-ion RT following systemic therapy remains limited, necessitating further investigation. To address this, we report a case series of eight advanced HCC patients who underwent C-ion RT after a favorable response to systemic therapy at our institution.

## Patients and methods

### Patients

**Fig. 1:**
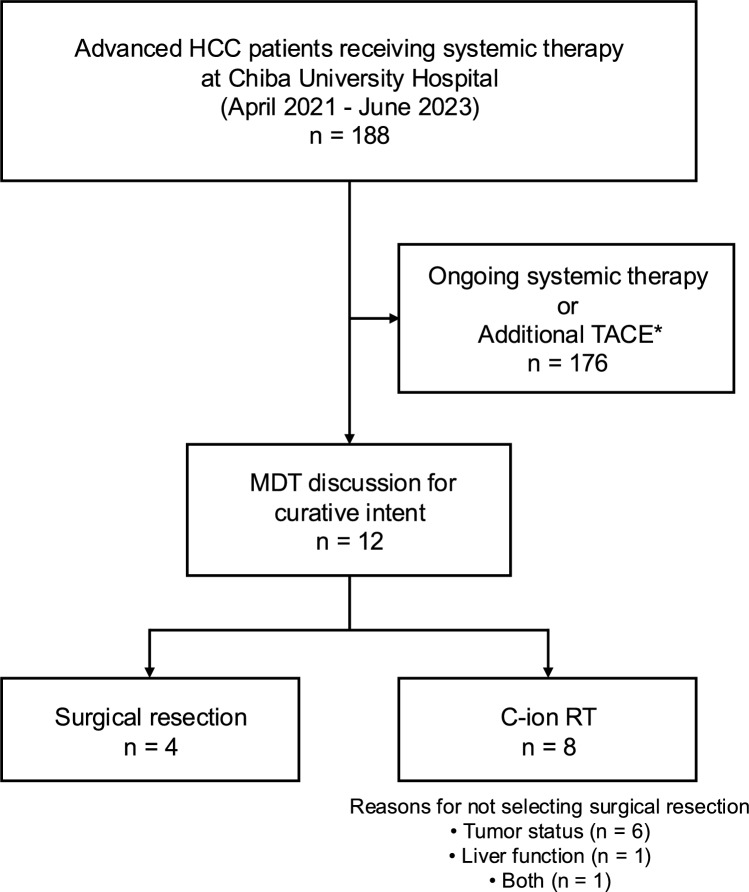
Patient selection and treatment allocation flowchart. This flowchart illustrates the patient selection process and treatment allocation among patients with advanced hepatocellular carcinoma (HCC) receiving systemic therapy at our institutions (April 2021 to June 2023). During this period, 188 patients received systemic therapy at our institutions. The majority (n = 176, 93.6%) continued systemic therapy alone or received additional transarterial chemoembolization (TACE) for local disease control (*Additional TACE refers to transarterial chemoembolization performed for local disease control while continuing systemic therapy, as distinct from curative-intent conversion therapy). 12 patients (6.4%) were evaluated through multidisciplinary team (MDT) discussion for curative-intent local therapy, resulting in surgical resection (n = 4, 2.1% of total cohort) or carbon ion radiotherapy (C-ion RT, n = 8, 4.3%). C-ion RT was selected for patients deemed unsuitable for surgical resection due to large tumor size (≥ 90 mm in 5/8 patients, 62.5%), major macrovascular invasion (5/8, 62.5%; including Vp4 in 3/8, 37.5%), impaired hepatic reserve (Child–Pugh B in 2/8, 25%), or unfavorable tumor location. These 8 consecutive cases represent all patients who underwent C-ion RT following systemic therapy during the study period. HCC, hepatocellular carcinoma; MDT, multidisciplinary team; TACE, transarterial chemoembolization; C-ion RT, carbon ion radiotherapy; MVI, macrovascular invasion; Vp4, portal vein invasion grade 4

This retrospective case series included eight patients with advanced HCC who underwent C-ion RT following systemic therapy at our institution and the National Institutes for Quantum Science and Technology (QST) between 2021 and 2023. During the study period, 188 patients with advanced HCC received systemic therapy at our institutions. Among these, 176 patients (93.6%) continued systemic therapy alone or received additional TACE for local disease control, while 12 patients (6.4%) were evaluated through multidisciplinary team discussion for curative-intent local therapy: surgical resection (n = 4, 2.1%) or C-ion RT (n = 8, 4.3%) (Fig. 1). These 8 cases represent all consecutive patients who received this treatment approach during the study period. Patients were selected for C-ion RT through multidisciplinary discussion, considering: (1) favorable tumor response to systemic therapy (tumor shrinkage, loss of contrast enhancement, or regression of vascular invasion); (2) absence of extrahepatic spread; (3) infeasibility of surgical resection due to poor liver function, insufficient remnant liver volume, or patient comorbidities; (4) adequate performance status and organ function; and (5) patient and family preference. Formal prospective selection criteria were not predefined due to the retrospective nature and evolving clinical practice. All patients received C-ion RT at 60 Gy (RBE) in 4 fractions, with no extrahepatic spread at the time of treatment. The interval between the last systemic therapy and C-ion RT initiation is summarized in Table 1.Data collection was completed in October 2024. This study was approved by the Research Ethics Committee of Chiba University (HK202309-02).

**Table 1:** Clinical features of our series

Case	Age	Sex	Etiology	Child–Pugh	Maximum size of intrahepatic tumor(mm)	Number of intrahepatic tumors	MVI	Treatment	Outcomes of medication	Sys → RT interval (days)
1	71	M	nBnC	5(A)	96.4	4	-	Atez/Bev	Tumor reduction	24
2	68	F	HBV	5(A)	29.7	1	Vp4	Atez/Bev	Disappearance of intrahepatic tumor Scar formation of tumor embolism	42
3	79	M	nBnC	6(A)	93	3	Vp4	Atez/Bev	Tumor reduction	56
4	67	M	nBnC(Alcohol)	8(B)	136	1	Vp4, Vv3	LEN	Tumor reduction	23
5	80	F	nBnC	5(A)	145	2	-	Atez/Bev	Tumor reduction	45
6	80	M	HBV	6(A)	100	2	Vp3	Atez/Bev	Disappearance of tumor enhancement	30
7	63	M	HBV	5(A)	41	6	-	Atez/Bev	Disappearance of tumor enhancement	61
8	70	M	HCV	7(B)	77	1	Vp2	Atez/Bev	Disappearance of tumor enhancement	30

Baseline clinical characteristics and systemic therapy details for eight patients with advanced hepatocellular carcinoma who underwent carbon ion radiotherapy following systemic therapy. Child-Pugh scores are shown in parentheses following the class designation. Macrovascular invasion (MVI) classification follows the Japanese staging system: Vp (portal vein invasion) grades 2–4 and Vv (hepatic vein invasion) grade 3. The Sys → RT interval indicates the time from the last administration of systemic therapy to the initiation of carbon ion radiotherapy.

HBV, hepatitis B virus; HCV, hepatitis C virus; MVI, macrovascular invasion; RT, radiotherapy; Sys, systemic therapy

### Clinical parameters

The baseline demographic data were retrospectively collected: gender, age, etiology, comorbidities, Eastern Cooperative Oncology Group performance status, Child–Pugh class, modified ALBI grade, radiological assessments, alpha-fetoprotein (AFP) levels, and prior treatment history. We also collected baseline demographics, adverse events (AEs), radiological progression dates, and survival data. Radiological evaluations were performed according to modified Response Evaluation Criteria in Solid Tumors (mRECIST). Adverse events were evaluated according to the Common Terminology Criteria for Adverse Events (CTCAE) version 5.0, including assessment for radiation-induced liver disease (RILD). Changes in ALBI scores over time were assessed descriptively due to the small sample size. No formal statistical testing was performed for efficacy endpoints given the exploratory nature of this case series.

## Results

### Patient characteristics

This study presents clinical data from 8 patients who underwent C-ion RT following systemic therapy (Table 1). The median age of the patients was 71 years (range 63–80), with six males and two females. The underlying liver disease included hepatitis B virus in three cases, hepatitis C virus in one case, and non-B, non-C (nBnC) in four cases. At the start of C-ion RT, liver function was classified as Child–Pugh A in 6 cases (score 5: 4 patients and score 6: 2 patients) and B in two (score 7: one patient and score 8: one patient). Maximum intrahepatic tumor diameter was ≥ 90 mm in five of eight patients. Prior to the initiation of C-ion RT, systemic therapy was administered in one case as lenvatinib and in seven cases as a combination of atezolizumab and bevacizumab. MVI was observed in 5 patients. Portal vein invasion was present in five patients (three with Vp4, one with Vp3, and one with Vp2), and hepatic vein invasion was present in one patient. One patient exhibited both portal vein and hepatic vein invasion. C-ion RT was selected as a radical treatment following favorable tumor response to systemic therapy, including tumor shrinkage, disappearance of intrahepatic lesions, regression of portal vein tumor thrombus, and loss of contrast enhancement. The median interval from the last systemic therapy to C-ion RT was 36 days (range, 23–61).

### Clinical outcomes

**Fig. 2:**
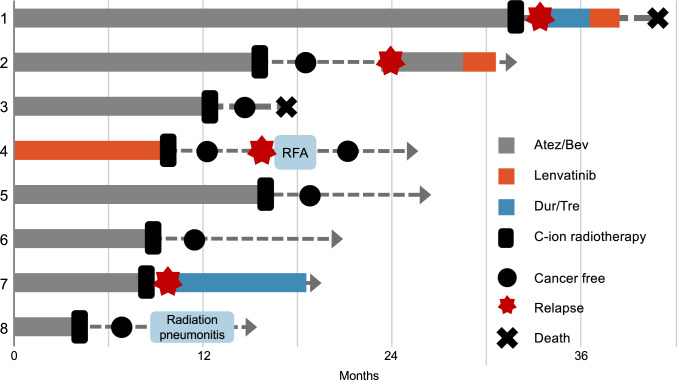
Swimmer plot of clinical courses for eight cases treated with carbon ion radiotherapy following systemic therapy. This figure illustrates the clinical progression of eight patients with advanced hepatocellular carcinoma treated with carbon ion radiotherapy after systemic therapy. Each bar represents the duration of treatment and follow-up for individual patients, highlighting key events such as recurrence, additional treatments, and adverse events. Cases achieving cancer-free status or requiring further interventions are noted

Figure 2 illustrates the individual clinical courses of all eight patients using a swimmer plot. Treatment details and clinical outcomes are summarized in Table 2. Four patients (cases 1, 2, 4 and 7) exhibited suspected recurrence during follow-up after C-ion RT and cases 1, 2, 7 were transitioned to systemic therapy. Two of these cases (1 and 7) developed new intrahepatic or distant metastases, while no in-field recurrence was observed. In case 7, lymph node metastasis manifested subsequent to C-ion RT, resulting in the resumption of systemic therapy. During follow-up, only one patient developed grade 1 radiation pneumonitis, which resolved without treatment. No cases of RILD, gastrointestinal bleeding, or severe hepatic toxicity were observed. The observation window for toxicity evaluation was from the initiation of C-ion RT to the last follow-up. AEs by case are summarized in Supplementary Table S1. Case 1 experienced intrahepatic recurrence soon after treatment and restarted systemic therapy, eventually dying due to tumor progression. Although Case 3 showed no recurrence after C-ion RT, the patient eventually died of empyema.

**Table 2:** Carbon ion radiotherapy treatment details and clinical outcomes

Case	C-ion RT dose/fractionation	EHS at C-ion RT	Follow-up duration (months)	rCF achieved	Recurrence after C-ion RT	Status
1	60 Gy (RBE)/4 fr	None	9.6	No	Yes (intrahepatic, 2 months)	DOD (9 months)
2	60 Gy (RBE)/4 fr	None	16.8	Yes	No	Alive, NED
3	60 Gy (RBE)/4 fr	None	5.3	Yes	No	Died of empyema (5 months)
4	60 Gy (RBE)/4 fr	None	16.4	Yes†	Yes (intrahepatic, 11 months)	Alive, treated with RFA
5	60 Gy (RBE)/4 fr	None	10.9	Yes	No	Alive, NED
6	60 Gy (RBE)/4 fr	None	12.4	Yes	No	Alive, NED
7	60 Gy (RBE)/4 fr	None	11.5	No	Yes (LN metastasis, 7 months)	Alive, on systemic therapy
8	60 Gy (RBE)/4 fr	None	11.6	Yes	No	Alive, NED

^†^Case 4 initially achieved rCF status (maintained for > 2 months) but later developed a solitary intrahepatic recurrence outside the irradiated field, which was successfully treated with radiofrequency ablation

Treatment parameters, follow-up duration, and clinical outcomes for eight patients with advanced hepatocellular carcinoma who underwent carbon ion radiotherapy (C-ion RT) following systemic therapy. All patients received C-ion RT with a uniform dose of 60 Gy (RBE) delivered in 4 fractions. None of the patients had extrahepatic spread (EHS) at the time of C-ion RT initiation. Follow-up duration is calculated from the completion of C-ion RT to the date of last follow-up or death (median 11 months, range 5–16 months). Radiological cancer-free (rCF) status was defined as the absence of viable intrahepatic lesions on contrast-enhanced imaging and no evidence of extrahepatic disease maintained for at least 2 months after C-ion RT, consistent with the criteria reported by Koroki et al. Five patients (62.5%) achieved rCF status. Recurrence timing (in parentheses) indicates time elapsed after C-ion RT completion.

C-ion RT, carbon ion radiotherapy; RBE, relative biological effectiveness; fr, fractions; EHS, extrahepatic spread; rCF, radiological cancer-free; NED, no evidence of disease; DOD, died of disease; LN, lymph node; RFA, radiofrequency ablation

**Fig. 3:**
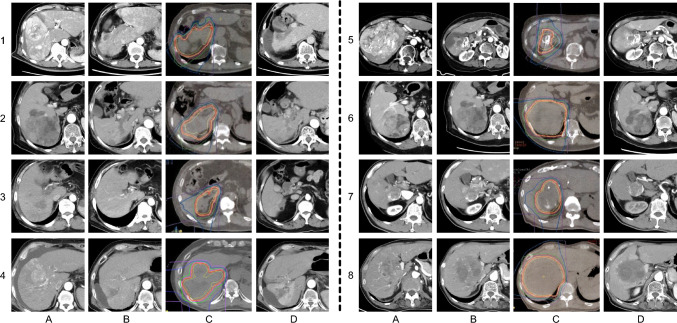
Serial radiological images of eight advanced hepatocellular carcinoma cases treated with C-ion RT. A: Baseline imaging at the initiation of systemic therapy. B: Imaging immediately prior to carbon ion radiotherapy (C-ion RT). C: C-ion RT treatment planning images. D: Follow-up imaging obtained 3–4 months after C-ion RT

**Fig. 4:**
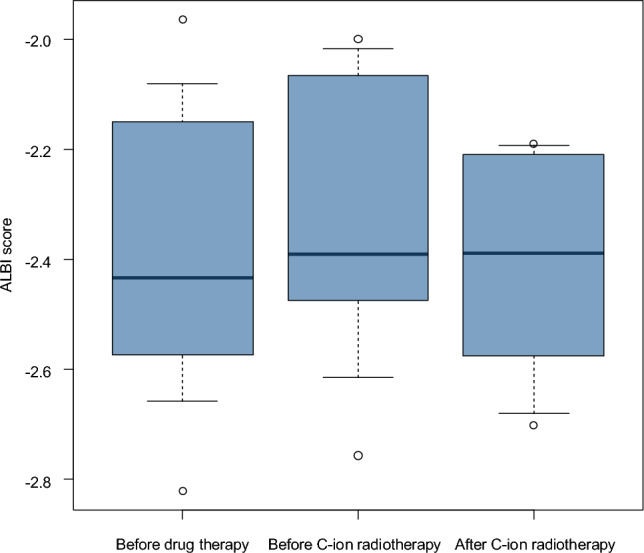
Box-and-whisker plot of ALBI scores before systemic therapy, before carbon ion radiotherapy, and after treatment. The box represents the interquartile range (IQR), the horizontal line within the box indicates the median, and the whiskers extend to the minimum and maximum values. ALBI scores remained stable throughout the treatment course, with no clinically meaningful deterioration in liver function observed, supporting the feasibility of C-ion RT in this setting

All eight patients received C-ion RT after systemic therapy. Five patients (cases 2, 3, 5, 6, and 8) demonstrated complete disappearance of contrast enhancement in all irradiated lesions and achieved rCF status (Fig. 3), defined as maintenance of recurrence-free status for at least 2 months after C-ion RT. Among these, cases 2, 5, 6, and 8 remained recurrence-free at the time of last follow-up, while case 3 died of empyema without evidence of HCC recurrence. In this study, rCF status was defined as the absence of viable intrahepatic lesions on contrast-enhanced imaging and no evidence of extrahepatic disease for at least 2 months after C-ion RT, consistent with the criteria reported by Koroki et al. [9]. This definition was chosen to capture early local control while acknowledging the limited follow-up duration of this exploratory series. The median follow-up duration after C-ion RT was 11.6 months (range, 5.3–16.8 months). Case 4 initially achieved rCF after C-ion RT but later developed a solitary extrahepatic recurrence, which was successfully treated with additional RFA. Cases 6, 7 and 8 also showed a decrease in tumor markers after C-ion RT. ALBI scores were measured at three time points—prior to systemic therapy, prior to C-ion RT, and after C-ion RT (Fig. 4)—and remained stable throughout the treatment course, with no clinically meaningful deterioration in liver function observed.

## Discussion

This exploratory case series provides preliminary evidence regarding the feasibility and short-term safety of C-ion RT following systemic therapy in a highly selected cohort of patients with advanced HCC. In this small series, C-ion RT was administered to patients who demonstrated favorable responses to systemic therapy but were deemed unsuitable for surgical resection due to poor liver function or technical limitations. Notably, C-ion RT was well tolerated in this context, with no significant decline in liver function observed during the limited follow-up period, even among elderly patients or those with Child–Pugh B cirrhosis.

In this study, five of eight patients had MVI, including three with main portal vein invasion (Vp4), and four of these five patients achieved rCF following C-ion RT. Additionally, four of five patients with tumors ≥ 90 mm in diameter achieved rCF, and recurrence-free status was maintained in four patients during the follow-up period. These outcomes suggest the feasibility and potential local effectiveness of C-ion RT even in patients with anatomically and biologically aggressive disease profiles. Notably, C-ion RT was able to achieve rCF status even in patients with Vp4, indicating that local control may be achievable despite the typically poor prognosis associated with MVI [12, 14]. Although two patients developed early recurrence after C-ion RT, these events represented new intrahepatic or distant metastases rather than local failure within the irradiated field. Therefore, local control was considered to have been achieved in all treated lesions. The prognostic value of rCF status in advanced HCC has been underscored in a recent large-scale study by Koroki et al., which demonstrated that patients who achieved rCF had significantly longer OS compared to those who did not (median OS not reached vs. 11.7 months; p < 0.001) [9]. Their findings validate rCF as a meaningful therapeutic milestone, and our results provide exploratory support for this concept. However, since only a highly selected subset of patients who responded favorably to systemic therapy underwent C-ion RT in this study, the present results should be interpreted cautiously and regarded as hypothesis-generating rather than confirmatory.

C-ion RT offers distinct physical and biological advantages over conventional radiotherapy. Its superior dose localization, enabled by the Bragg peak phenomenon and high linear energy transfer, allows for precise targeting of tumors while sparing surrounding normal liver tissue [10]. In our cohort, no significant treatment-related toxicities were observed, even in patients requiring large radiation fields. This favorable safety profile is particularly relevant in advanced HCC, where liver function is often marginal and dose limitations hinder the use of traditional X-ray radiation therapy [15, 16]. In advanced HCC, MVI often coexists with large tumor burden, both of which present significant challenges for curative treatment. Giant tumors are frequently associated with MVI and anatomical complexity, making surgical resection technically difficult and often contraindicated, particularly in patients with poor liver function. In this context, the ability of C-ion RT to provide high-dose, localized treatment with minimal impact on surrounding liver parenchyma appears to be of clinical interest. Our results demonstrated that C-ion RT was safely administered to patients with large tumors, achieving tumor control in some cases without deterioration in liver function. These findings suggest that C-ion RT could be a feasible local modality for selected patients, consistent with previous reports showing favorable local control rates with C-ion RT in bulky or MVI-positive HCC [10, 11].

Despite the encouraging results, this study has several important limitations that warrant careful interpretation. First, the sample size is very small (n = 8), limiting statistical power and generalizability. Second, the median follow-up period of 11 months is insufficient to assess long-term outcomes, including overall survival and late toxicities. Third, the retrospective, single-center design introduces potential selection bias. As shown in Fig. 1, C-ion RT was reserved for patients unsuitable for surgical resection, representing only 4.3% of all patients receiving systemic therapy (8/188) and 66.7% of those selected for curative-intent local therapy (8/12) during the study period. Patients were selected through multidisciplinary discussion without predefined prospective criteria, and only those who responded favorably to systemic therapy and were deemed suitable candidates for local consolidation were included. This approach inherently selects for patients with more advanced local disease characteristics, including larger tumors (≥ 90 mm in 62.5%), higher rates of main portal vein invasion (Vp4 in 37.5%), and poorer hepatic reserve (Child–Pugh B in 25%) compared to patients selected for surgical resection. This limits the generalizability of our findings to broader patient populations and precludes direct comparison with outcomes of surgical resection without appropriate adjustment for these baseline differences. Fourth, the lack of a control group precludes definitive conclusions regarding the incremental benefit of C-ion RT over continued systemic therapy alone or observation. Therefore, these findings should be interpreted as exploratory and hypothesis-generating rather than confirmatory, and serve primarily to provide a rationale for future prospective studies with standardized patient selection criteria, longer follow-up, and appropriate control groups.

In the current era of highly effective systemic therapies, including immune checkpoint inhibitors, the concept of conversion therapy has gained increasing relevance [7]. As more patients achieve partial or complete responses to systemic treatment, the integration of locally directed treatments such as C-ion RT may offer a pathway toward sustained disease control in selected cases. While our study suggests that achieving rCF through C-ion RT is feasible and may contribute to favorable outcomes, these findings are preliminary and require confirmation in larger cohorts. C-ion RT should therefore be regarded as a promising and feasible component of multidisciplinary management for patients with advanced HCC who respond to systemic therapy, rather than an established curative option at present. As systemic therapies continue to evolve, the role of C-ion RT in achieving durable local control and improving long-term outcomes may become increasingly significant.

## Supplementary Information

Below is the link to the electronic supplementary material.

Supplementary file1

## Data Availability

The data supporting the findings of this study are available from the corresponding author upon reasonable request.

## Abbreviations

ABC: Atezolizumab plus bevacizumab conversion
AFP: Alpha-fetoprotein
ALBI: Albumin-bilirubin
BCLC: Barcelona clinic liver cancer
C-ion RT: Carbon ion radiotherapy
CTCAE: Common terminology criteria for adverse events
DFS: Disease-Free survival
EHS: Extrahepatic spread
HBV: Hepatitis B virus
HCC: Hepatocellular carcinoma
HCV: Hepatitis C virus
LET: Linear energy transfer
MVI: Macrovascular invasion
OS: Overall survival
PFS: Progression-free survival
RFA: Radiofrequency ablation
mRECIST: Modified response evaluation criteria in solid tumors
rCF: Radiological cancer-free
TACE: Transarterial chemoembolization
