# Supplementary material for: Exploratory case series of conversion to carbon ion radiotherapy after systemic therapy in advanced hepatocellular carcinoma
Source: Int Cancer Conf J. 2026 Jan 12;15(2):195–202. doi: 10.1007/s13691-025-00839-x (PMC13038779; doi:10.1007/s13691-025-00839-x)
Supplement: Supplementary file 1 — Supplementary file1 [file 13691_2025_839_MOESM1_ESM.docx]

**Supplementary Table Legend**

**Supplementary Table S1. Adverse events during and after C-ion RT (CTCAE v5.0).**

Adverse events following carbon-ion radiotherapy (C-ion RT) after systemic therapy in eight patients with advanced hepatocellular carcinoma. Toxicities were graded according to CTCAE version 5.0. Radiation-induced liver disease (RILD) was evaluated according to classic and non-classic definitions (classic: anicteric hepatomegaly, ascites, and elevated alkaline phosphatase ≥2× upper limit of normal within 4 months; non-classic: ≥5-fold elevation in ALT/AST within 3 months in absence of progression). The observation window for toxicity evaluation was from the initiation of C-ion RT to the last follow-up.

Abbreviations: C-ion RT, carbon ion radiotherapy; CTCAE, Common Terminology Criteria for Adverse Events; RILD, radiation-induced liver disease; RT, radiotherapy.

**ssTable S1.** Adverse events during and after C-ion RT (CTCAE v5.0)

| Case | Adverse event(s) | CTCAE grade | RILD | Time window | Outcome |
| --- | --- | --- | --- | --- | --- |
| 1 | None | – | None | – | – |
| 2 | None | – | None | – | – |
| 3 | None | – | None | – | – |
| 4 | None | – | None | – | – |
| 5 | None | – | None | – | – |
| 6 | None | – | None | – | – |
| 7 | Radiation pneumonitis | Grade 1 | None | 3 months post-RT | Resolved without treatment |
| 8 | None | – | None | – | – |

Abbreviations: CTCAE, Common Terminology Criteria for Adverse Events; RILD, radiation-induced liver disease
